# Supplementary material for: Unveiling the Genomic Features and Biocontrol Potential of Trichoderma hamatum Against Root Rot Pathogens
Source: J Fungi (Basel). 2025 Feb 8;11(2):126. doi: 10.3390/jof11020126 (PMC11856919; doi:10.3390/jof11020126)
Supplement: Supplementary file 1 [file jof-11-00126-s001.zip › Supplementary Materials1.pdf]

Table S1. Antagonistic activity of *Trichoderma hamatum* against three *Fusarium* pathogenic fungi. No significant differences were observed among the inhibition rates of *T. hamatum* against the three *Fusarium* pathogenic fungi (*P* > 0.05, based on ANOVA followed by LSD test).

Strain name	Inhibition rate against *F. oxysporum* (%)	Inhibition rate against *F. solani* (%)	Inhibition rate against *F. acutatum* (%)
T. hamatum	70.63±3.63	68.07±2.74	66.12±3.63

Table S2. HiFi data statistics.

data_type	Total base (Gbp)	Reads Numbers	N50 (bp)	Depth (X)
HiFi	6.81995	393,002	17,788	162.646

Table S3. Base composition and GC content in the genome.

	Length (bp)	% of genome
A	11,239,667	26.81
T	11,247,593	26.82
C	9,719,027	23.18
G	9,724,843	23.19
N	0	0
GC	19,443,870	46.37
total	41,931,130	100

Table S4. Sequencing, assembly metrics, and genome quality of *T. hamatum.*

Assembly	*T. hamatum*
Number of contigs	31
Assembly length (bp)	41,931,130
Contig N50 (bp)	6,071,830
HiFi reads mapping rate (%)	99
HiFi reads coverage (%)	99.98
BUSCO (%)	98.7
QV	62.3888

Table S5. Basic statistics of gene prediction.

Gene set	Number	Average gen length (bp)	Average CDS length (bp)	Average exon per gene	Average exon length (bp)	Average intron length (bp)
denovo/AUGUSTUS	9814	1956.07	1671.91	2.96	565.41	145.2
denovo/GlimmHMM	11611	1732.65	1458.29	2.59	563.28	172.67
homo/T.harzianum	12057	1776.35	1236.51	2.45	505.72	373.58
homo/T.reesei	10745	1770.84	1236.37	2.45	504.86	368.87
homo/T.atroviride	11659	1817.64	1261.73	2.47	510.3	377.53
homo/T.virens	11055	1789.94	1256.97	2.5	503.22	355.82
MAKER	10774	1737.61	1537.48	2.73	563.32	117.05

Table S6. Functional annotation statistics

	Number	Percent (%)
Total	10774	
Annotated	10764	99.91
InterPro	8242	76.5
GO	7922	73.53
KEGG_ALL	10575	98.15
KEGG_KO	4164	38.65
Swissprot	7065	65.57
TrEMBL	10758	99.85
NR	10764	99.91
Unannotated	10	0.09

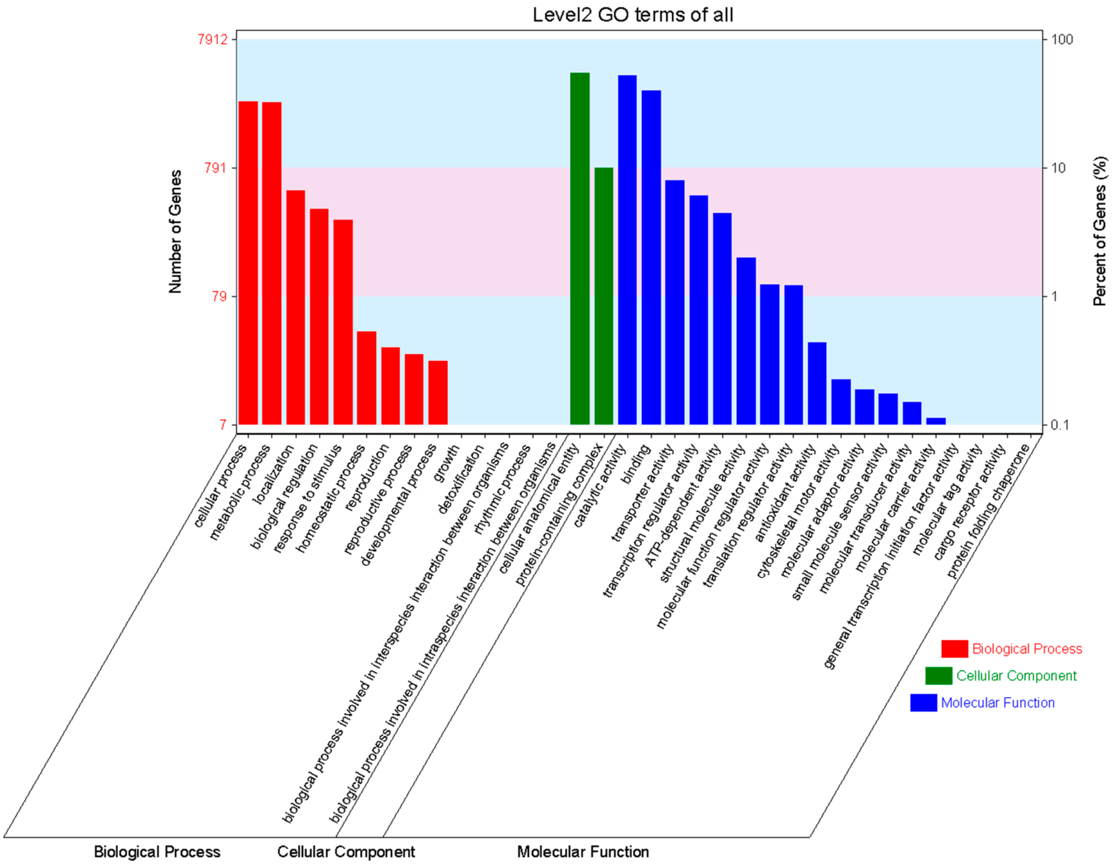

Figure S1. Statistical map of functional annotation classification based on GO database.

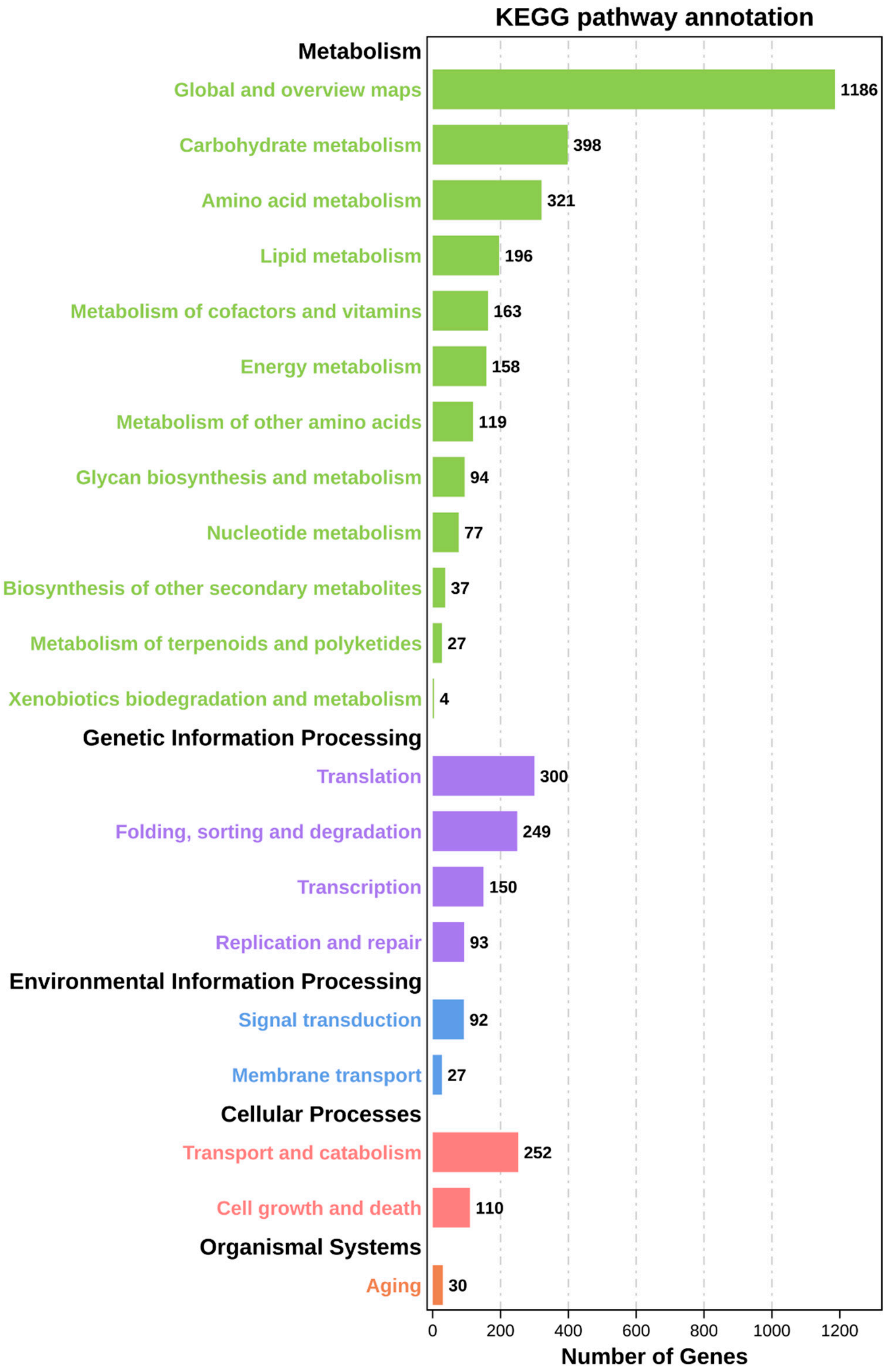

Figure S2. KEGG Pathway Annotation of Coding Sequences in the Whole Genome of *T. hamatum*.

The black font on the y-axis represents the first-level KEGG pathways, while the text below indicates the second-level KEGG pathway classifications. The x-axis displays the number of genes annotated in each classification.

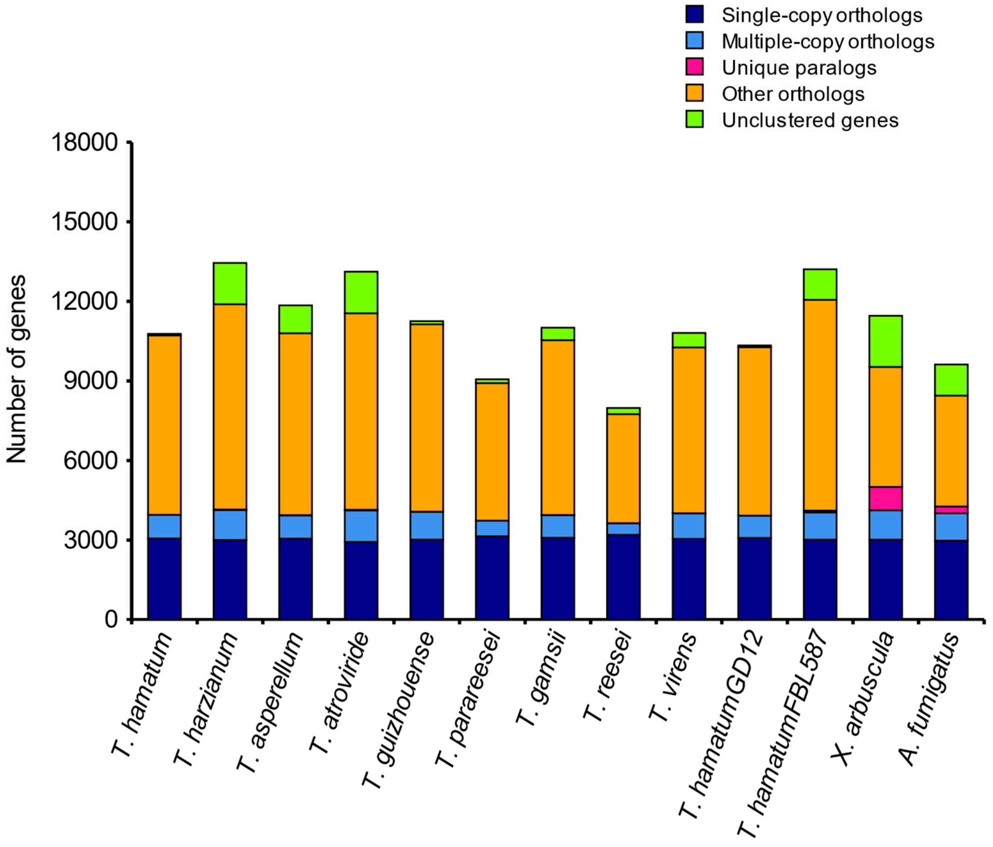

Figure S3. Comparative genomic analysis of *T. hamatum* and twelve strains. This figure illustrates the number of genes present in various strains, comparing *T. hamatum* with twelve other strains.

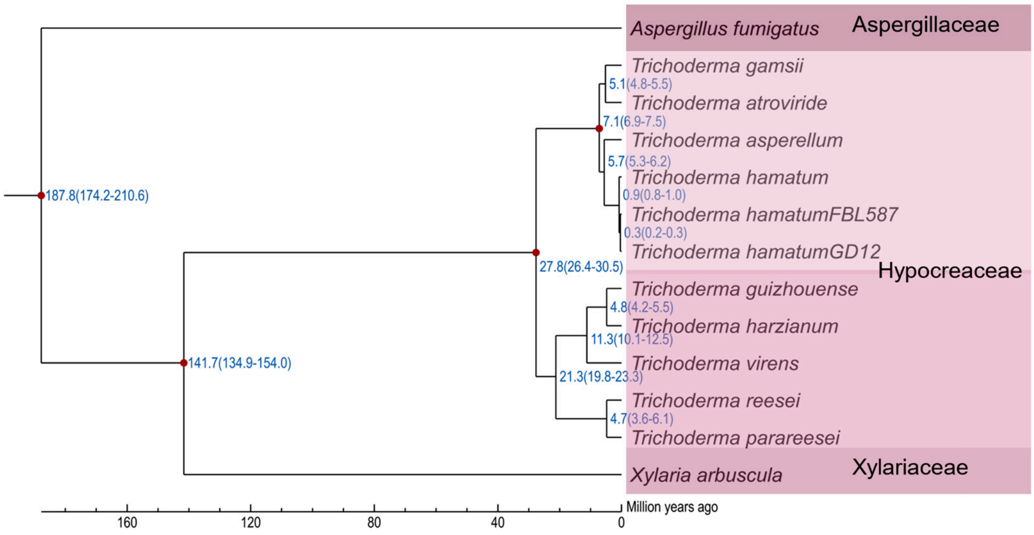

Figure S4. Estimated species divergence times. The numbers at the node positions indicate the estimated divergence times, while the numbers in parentheses represent the confidence intervals for these divergence times, measured in millions of years. Red nodes denote the time calibration points.

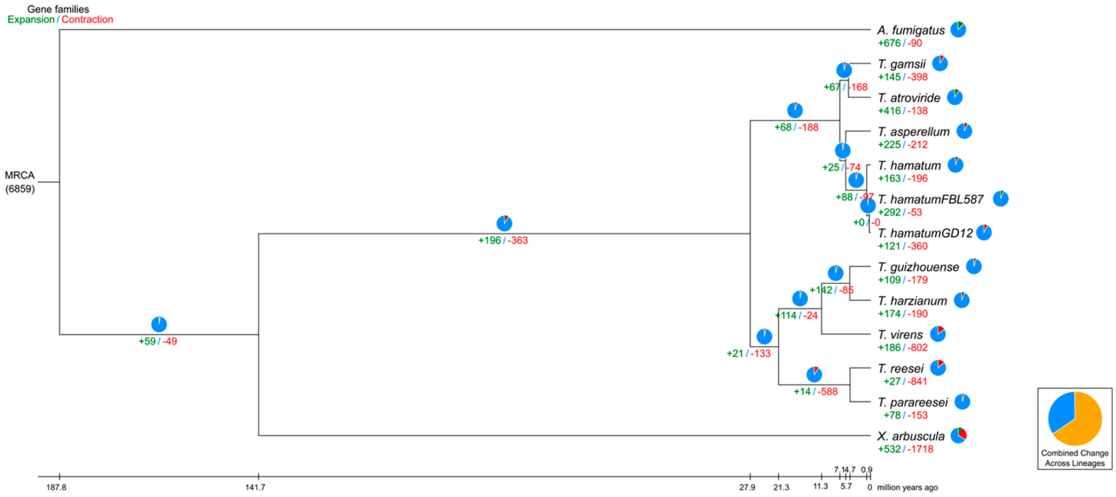

Figure S5. Illustrates the phylogenetic tree along with information regarding gene family expansions and contractions. Green numbers indicate the number of expanded gene families, while red numbers denote the number of contracted gene families.

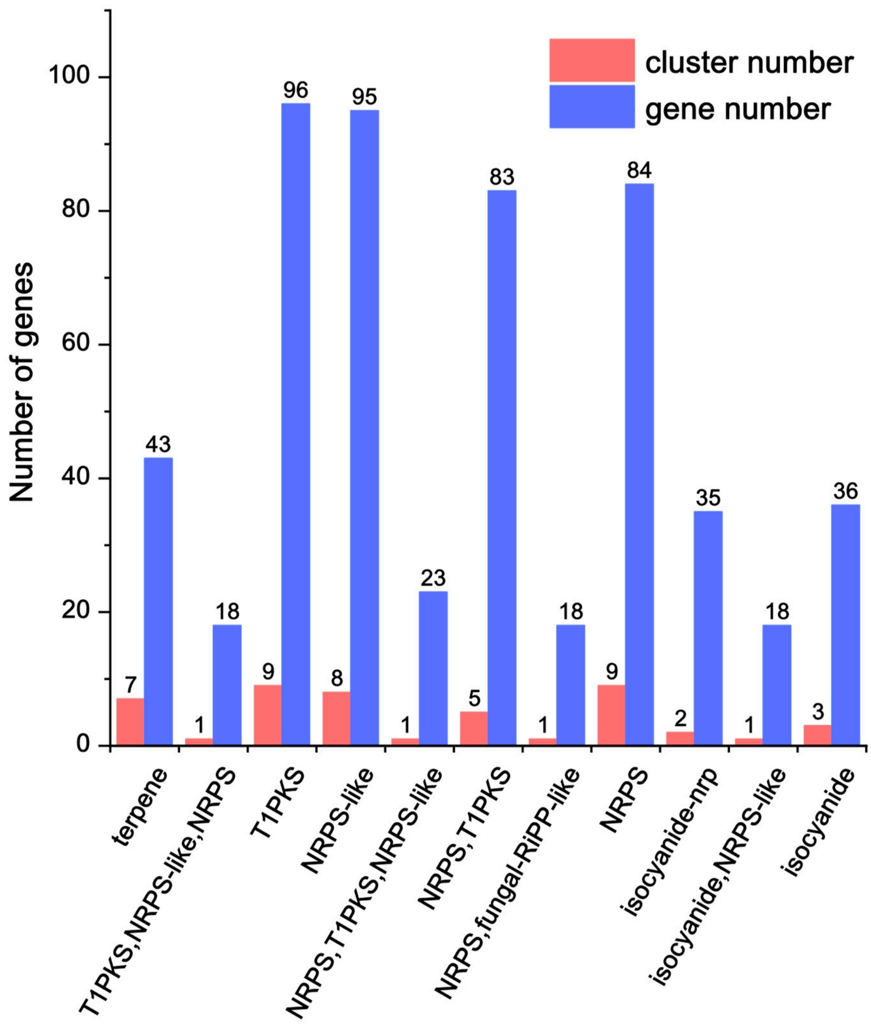

Figure S6. Quantitative analysis of secondary metabolite genes. The red bars indicate the number of gene clusters, while the blue bars represent the number of genes involved in biosynthesis in *T. hamatum*. T1PKS: Type I Polyketide Synthase; NRPS: Non-Ribosomal Peptide Synthetase; RIPP: Ribosomally Synthesized and Post-Translationally Modified Peptides; terpene: Terpenes.

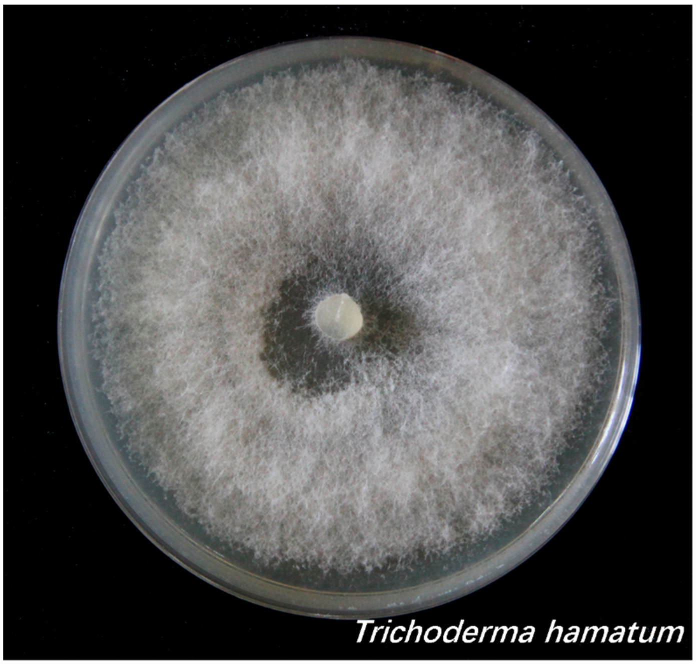

Figure S7. The blank control of *T. hamatum* cultured alone.
